# Atrial Tachycardia Originating from the Cavo-Tricuspid Isthmus May Exhibit Narrow P Waves

**Published:** 2010-03-05

**Authors:** Takumi Yamada, H. Thomas McElderry, James D Allred, Harish Doppalapudi, G. Neal Kay

**Affiliations:** Division of Cardiovascular Disease, University of Alabama at Birmingham, Birmingham, AL, USA

**Keywords:** focal atrial tachycardia, cavo-tricuspid isthmus, P wave, radiofrequency catheter ablation

## Abstract

An 83-year-old man underwent electrophysiological testing for focal atrial tachycardia (AT) exhibiting narrow P waves with negative deflections in the inferior leads. Catheter ablation at the cavo-tricuspid isthmus (CTI) successfully eliminated the AT. The propagation map during AT and pacing study from the successful ablation site demonstrated that the atrial activation throughout the CTI did not produce significant P wave deflections. Consequently, during AT, the left atrial activation time determined the P wave duration. This case demonstrates that AT originating from the CTI may exhibit narrow P waves which can be misinterpreted as AT originating from the inter-atrial septum.

## Introduction

Focal atrial tachycardias (ATs) originating from either the right atrium (RA) or left atrium (LA) have been proved to be good candidates for radiofrequency (RF) catheter ablation. Although electroanatomic mapping may be helpful for identifying the exact site of an AT origin, the P wave morphology is a useful clue during electrophysiological study because it can predict the site of the AT origin easily and non-invasively [1,2]. However, algorithms using the P wave morphology to predict the site of the AT origin can be limited by the specific atrial anatomy. We report a case with an AT which may be an exception to these algorithms.

## Case

**Figure 1:**
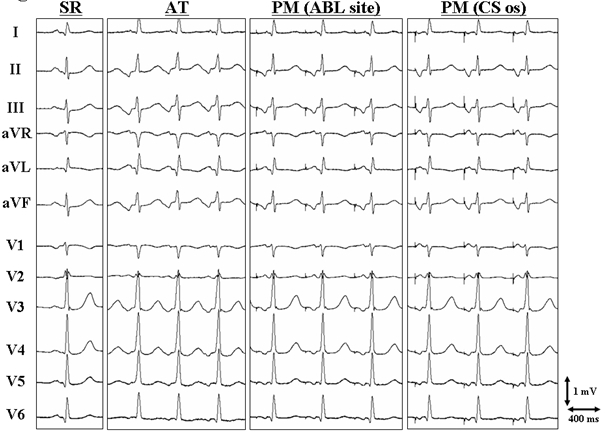
Twelve-lead electrocardiograms during sinus rhythm (SR), atrial tachycardia (AT) and pacing from the successful ablation (ABL) site and ostium of the coronary sinus (CS os). PM=pace map.

**Figure 2:**
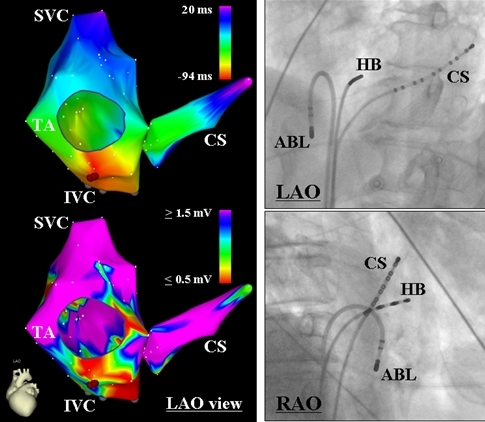
Activation and voltage maps of the right atrium and coronary sinus (CS) during the tachycardia (left panels) and fluoroscopic images exhibiting the successful ablation site (right panels). In the activation map, the red indicates the areas with the earliest endocardial activation and orange, yellow, green, blue and purple indicate a progressively delayed activation while in the voltage map, the purple and red indicate the areas with a voltage of the local bipolar electrogram >= 1.5 mV and <= 0.5 mV, respectively. The red tags indicate the successful ablation site. HB=His bundle; IVC=inferior vena cava; LAO=left anterior oblique; RAO=right anterior oblique; SVC=superior vena cava; TA=tricuspid annulus. The other abbreviations are as in Figure 1.

**Figure 3:**
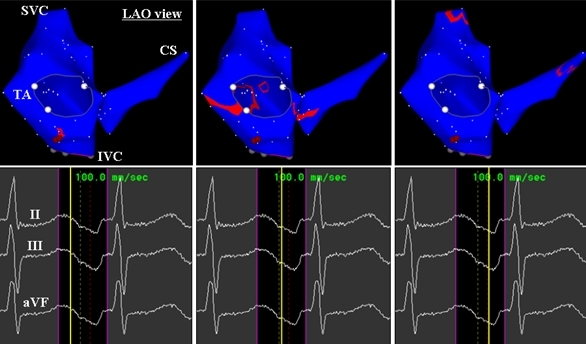
Propagation maps of the right atrium and CS during the tachycardia. The upper panels show the propagation maps and lower panels the timing on the surface electrocardiogram corresponding to the propagation map (solid yellow line). The other abbreviations are as in figure 2.

An 83-year-old man with coronary artery disease underwent an electrophysiological study (EPS) and catheter ablation for supraventricular tachycardia (SVT). Echocardiography revealed no evidence of structural heart disease. Written, informed consent was obtained, and the EPS was performed after all antiarrhythmic drugs had been discontinued for at least five half-lives prior to the study. At baseline, the SVT was incessant. During sinus rhythm the 12-lead electrocardiogram exhibited normal P waves. The SVT was a long R-P' tachycardia (cycle length = 520 ms) with narrow P waves that were negative in the inferior leads and biphasic (+/-) in lead V1 (Figure 1). Multipolar catheters were introduced via the right common femoral vein and positioned in the coronary sinus (CS), His bundle region and right ventricular apex for mapping and pacing. During sinus rhythm, the AH and HV intervals were normal. Burst ventricular pacing during the SVT revealed V-A dissociation. Activation mapping with a 7-French, 4-mm tip non-irrigated ablation catheter (NAVI-STAR™ EZ STEER™, Biosense Webster, Diamond Bar, CA, USA) was then performed in the RA and CS during the SVT. It revealed a centrifugal activation pattern from the middle cavo-tricuspid isthmus at 6 o'clock around the tricuspid annulus (Figure 2) and a definite diagnosis of an atrial tachycardia (AT) was made. It was noted that the top of the RA and postero-lateral portion of the CS were activated simultaneously (Figure 2). A radiofrequency application was delivered at the site of earliest atrial activation and successfully eliminated the AT. Pacing from the successful ablation site exhibited an isoelectrical segment between the pacing stimulus artifact and the onset of the P wave and reproduced a perfect match to the narrow P waves of the AT (Figure 1). Though atrial pacing from the CS ostium exhibited similar P waves to those during the AT and pacing from the site of the AT origin, the duration of the P wave was shorter during the AT and pacing from the site of AT origin than during pacing from the CS ostium (Figure 1). The propagation map during the AT revealed that the atrial activation from the AT origin to the CS ostium and lower RA free wall did not produce a significant P wave deflection on the surface electrocardiogram whereas the activation from those sites toward the superior portion of the atria was responsible for the P wave (Figure 3). The voltage map revealed that the whole area of the cavo-tricuspid isthmus exhibited local atrial electrograms of lower voltage as compared with those in the other RA sites (Figure 2).

## Discussion

It is known that the sites of AT foci are usually associated with the specific anatomic locations [1,2]. In the RA, the sites along the tricuspid annulus are one of the major sources of AT foci [3,4]. The AT origin in this case was located at the cavo-tricuspid isthmus as a part of the sites along the tricuspid annulus.

Algorithms using the P wave morphology to predict the site of the AT origin should be consistent with the anatomical considerations. In general, ATs with an origin in the atrial free wall should exhibit broader P waves than those with an origin in the inter-atrial septum (IAS) because activation from the AT origin in the IAS can propagate throughout the atrium faster than that from the AT origin in the atrial free wall. However, in this case P waves appeared to be narrower during AT and pacing from the site of the AT origin than during pacing from the CS ostium. A previous study demonstrated that during cavo-tricuspid isthmus dependent atrial flutter, the atrial activation which goes through the cavo-tricuspid isthmus represents the isoelectrical segment between the negative saw-tooth waves in the inferior leads while the atrial activation in the LA and IAS represents the negative saw-tooth waves in the inferior leads [5]. In fact, in this case the propagation map during the AT and pacing study from the successful ablation site demonstrated that the atrial activation which went through the cavo-tricuspid isthmus did not produce any significant P wave deflections on the surface electrocardiogram. Consequently, during the AT, the activation of the LA and rest of the RA was responsible for the surface P wave deflections and the duration of P waves was determined by the activation time in the LA which was longer than that in the rest of the RA. The activation time in the LA should have been identical between the AT and pacing from the CS ostium in this case. Therefore, in this case the activation time in the RA was suggested to be longer than that in the LA during pacing from the CS ostium because otherwise, the AT and pacing from the CS ostium would have exhibited P waves with the same duration. The isoelectrical segment in the inferior leads during the AT in this case may have been produced by vectorial expression of activation and/or slow and low voltage activation in the cavo-tricuspid isthmus. This case demonstrates that an AT originating from the cavo-tricuspid isthmus may exhibit narrow P waves which can be misinterpreted as an AT originating from the IAS.
